# School Clinics in London

**Published:** 1910-05-21

**Authors:** 


### The Hospital

A Journal of

The Medical Sciences and Hospital Administration.

New Series. No. 170, Vol. VII. [No. 1242, Vol. XLVIII.] SATURDAY, MAY 21, 1910.

### SCHOOL CLINICS IN LONDON.

Amid the temporary national distraction from economic and social questions caused by the lamented decease of King Edward, there is some chance that the medical profession may fail at once to appreciate the very important aims and resolutions of the Metropolitan Counties Branch of the British Medical Association, as expressed at the Conference held on May 4, at St. James's Vestry Hall. As the Chairman (Dr. Ford Anderson) said, the present is the only time at which any action is likely to be successful before custom, precedent, and vested interests have time to set themselves up as obstacles to the establishment of correct principles of advance. At this juncture the line of action for the whole of the future rests upon the decision of the medical profession, because what the profession decides, the public, as represented by its municipal bodies, must accept. There is this much to be said in qualification of Dr. Anderson's remarks, that political factors and jealousies are also to be taken into account. It is well known that the present County Council, like almost every preceding Council, is at variance with the Government of the day, because London, for some mysterious reason that nobody can fathom, takes care to return a Municipal Reform Council when the Radicals are in power at Westminster, and a Progressive Council when the Conservatives rule the country. The result of this curious fact is that there is generally very little love lost between Spring Gardens and Whitehall, and political antagonisms and controversies are allowed to interfere with the solution of questions which should properly be outside politics altogether. In the present case the Municipal Reformers are holding office because, four years ago, London concluded, rightly or wrongly does not matter, that the other side had been spendthrift and extravagant; and accordingly they seek to justify their return by the most careful economy. The Government, on the other hand, is supported by many members who were of the defeated Progressives, and even contains the ex-leader of that faction. Accordingly, it is not at all averse to a policy which will increase municipal expenditure in London, for thus it hopes to discredit the Municipal Reformers by preventing their promised economies, while at the same time it relieves the national exchequer and the embarrassments of its Chancellor.

The upshot of this unfortunate state of things is that the London County Council, faced with the permissive legislation on the inspection of school children, seeks the least expensive way to carry out its duties, because it is well known that the minority, while still backing every scheme which will increase municipal expenditure, is eager enough to go to the electorate on the cry of their opponents' broken pledges. Thus a cheeseparing policy is practically forced upon the Municipal Reformers by political exigencies. The easiest way out of the difficulty of inspecting and treating school children without running up a big bill is to sweat the medical profession and to impose on the voluntary hospitals maintained by the charitable; and that accordingly is what the Education Committee, by a narrow majority, decided a year ago to do. The committee is, of course, not in the least to blame for this decision. If the profession and the hospitals are foolish enough to do other people's duties for nothing, so much the worse for them; their folly relieves the ratepayers of London, and incidentally allows one political faction to score off the other. But if it can be shown that the arrangement is a workable means of securing the desired ends; if it can be shown that it does not unduly crowd the hospitals, does not deprive the general practitioner of work and fees which he might otherwise earn, does not involve the alienation of hospital subscriptions from the purposes for which they were intended, then the Education Committee is to be congratulated on the solution of a difficult problem.

This is, to all intents and purposes, the point which was debated at the Metropolitan Counties Branch meeting. With three dissentients, it was decided that "the provisional arrangements made by the London County Council for the medical treatment of school children are wholly inadequate, and involve abuses of charitable medical institutions and unfairness to the medical profession"; and unanimously that "provision should be made by the London County Council for the medical treatment of school children at suitable centres by local medical practitioners." It is to be presumed that the three dissentients refrained from voting to the second resolution, and that they were in fact the three members of hospital staffs who spoke during the discussion. One of these gentlemen, who advocated his views with energy, was subjected to a great deal of interruption during his speech; he concluded with the remark that the resolutions are entirely premature, and that the arrangements of the County Council involve no abuse. The voting upon these resolutions was so decisive that there is no possible doubt as to the opinion of the members of the British Medical Association in and around London upon this matter. The second resolution to all intents amounts to a demand for the establishment of school clinics, and this is the rock upon which the whole Conference may come to shipwreck. Whether or not the free treatment of school children by the State or the municipality is desirable and imperative is one question; whether or no as practical politics school clinics financed by the London County Council are feasible is a totally different proposition, and one which medical practitioners will do well to study in the light of what has already been said about the division of parties on the Council. Party faction is no doubt most regrettable in these matters, and for that reason alone it is perhaps unfortunate that the mover of these resolutions and several of his prominent supporters should be members of the extreme wing of a party which is regarded, not unnaturally, with suspicion and distrust by the present majority at Spring Gardens. It is to be hoped that some way may be found by which the interests of the children, the due remuneration of the medical profession for work done, and the utmost possible saving of the public purse may all be reconciled; but no scheme propounded so far combines these advantages, and certainly the resolutions passed on May 4 are not very likely to further such a desirable consummation.

